# From Inquilines to Gall Inducers: Genomic Signature of a Life-Style Transition in *Synergus* Gall Wasps

**DOI:** 10.1093/gbe/evaa204

**Published:** 2020-09-28

**Authors:** Erik Gobbo, Nicolas Lartillot, Jack Hearn, Graham N Stone, Yoshihisa Abe, Christopher W Wheat, Tatsuya Ide, Fredrik Ronquist

**Affiliations:** Department of Bioinformatics and Genetics, Swedish Museum of Natural History; CNRS, Laboratoire de Biométrie et Biologie Evolutive UMR 5558, Université de Lyon,France; Vector Biology Department, Liverpool School of Tropical Medicine; Institute of Evolutionary Biology, University of Edinburgh; Biosystematics Laboratory, Faculty of Social and Cultural Studies, Kyushu University; Department of Zoology, Stockholm University; Department of Zoology, National Museum of Nature and Science, Amakubo, Tsukuba; Department of Bioinformatics and Genetics, Swedish Museum of Natural History

**Keywords:** codon models, Bayesian selection analysis, gene set enrichment analysis, gene duplication, gall induction, Cynipidae

## Abstract

Gall wasps (Hymenoptera: Cynipidae) induce complex galls on oaks, roses, and other plants, but the mechanism of gall induction is still unknown. Here, we take a comparative genomic approach to revealing the genetic basis of gall induction. We focus on *Synergus itoensis*, a species that induces galls inside oak acorns. Previous studies suggested that this species evolved the ability to initiate gall formation recently, as it is deeply nested within the genus *Synergus*, whose members are mostly inquilines that develop inside the galls of other species. We compared the genome of *S. itoensis* with that of three related *Synergus* inquilines to identify genomic changes associated with the origin of gall induction. We used a novel Bayesian selection analysis, which accounts for branch-specific and gene-specific selection effects, to search for signatures of selection in 7,600 single-copy orthologous genes shared by the four *Synergus* species. We found that the terminal branch leading to *S. itoensis* had more genes with a significantly elevated d*N*/d*S* ratio (positive signature genes) than the other terminal branches in the tree; the *S. itoensis* branch also had more genes with a significantly decreased d*N*/d*S* ratio. Gene set enrichment analysis showed that the positive signature gene set of *S. itoensis*, unlike those of the inquiline species, is enriched in several biological process Gene Ontology terms, the most prominent of which is “Ovarian Follicle Cell Development.” Our results indicate that the origin of gall induction is associated with distinct genomic changes, and provide a good starting point for further characterization of the genes involved.

**Significance**

Gall wasps are a family of gall-making plant parasite. How exactly they are able to make said galls is still unknown. We identified several genes that might be involved in the process by comparing the genomes of a true gall-maker with that of three related wasps that are unable to initiate a gall in their host plant.

## Introduction

Gall wasps (Hymenoptera: Cynipidae) are plant parasites that induce the development of highly modified plant tissues, termed galls, in which their immature stages feed and grow (Abrahamson and Melika 2002; Stone et al. 2002). The cynipid larva is enclosed inside a gall chamber lined with specialized nutritive cells formed by the plant (Stone and Schönrogg0065 2003; Harper et al. 2004; Hearn et al. 2019). Although all cynipids appear able to induce the development of such nutritive tissues, several lineages—termed inquilines—can only induce nutritive tissue development within galls of other species (Sanver and Hawkins 2000). The inquilines can thus be seen as cynipids that induce a “gall within a gall.” The presence of inquilines can negatively affect the fitness of the primary gall inducer, in some cases killing them (László and Tóthmérész 2006).

Several hypotheses on the mechanism of cynipid gall induction have been advanced, partly inspired by knowledge of other gall-inducing organisms: Secretion of auxins (Tooker and Helms 2014), injection of virus-like particles (Cornell 1983; Cambier et al. 2019), manipulation of plant NOD factors (Hearn et al. 2019), or involvement of bacterial or fungal symbionts (Hearn et al. 2019). However, in contrast to some other gall induction systems (Harris and Pitzschke 2020), there is no conclusive evidence for any of these hypotheses in cynipids. A phylogenomic approach should provide, if not an answer, then at least a clue of where to find one. Diversity of a trait of interest in closely related species allows us to use selection analysis to identify genetic factors associated with evolutionary changes in the trait (Suzuki 2010). These methods are based on comparison of the frequencies of nonsynonymous (d*N*) and synonymous (d*S*) mutations in coding sequences: A low d*N*/d*S* ratio (<1.0) suggests purifying selection, which is expected in genes of conserved function, whereas high ratios (>1.0) suggest positive selection consistent with a signature of rapid adaptation to a changing function. Even if ratios >1.0 are likely to be rare, the origin of a trait of interest should be accompanied by increased d*N*/d*S* ratios in the genes coding for the trait compared with background levels (Yang and Nielsen 2002). It is, however, worth noting that these kind of methods are subject to many errors (Markova-Raina and Petrov 2011), and the implementation of many levels of control is recommended. Given that such controls are implemented, and that we can place the acquisition of the trait in a phylogenetic tree, we can search for changes in selection pressure associated with particular genes on that branch of the tree. To use selection analysis to identify genes associated with the origin of gall induction in cynipids, we thus need to pinpoint where on the tree the ability to induce galls evolved.

**Fig. 1.**
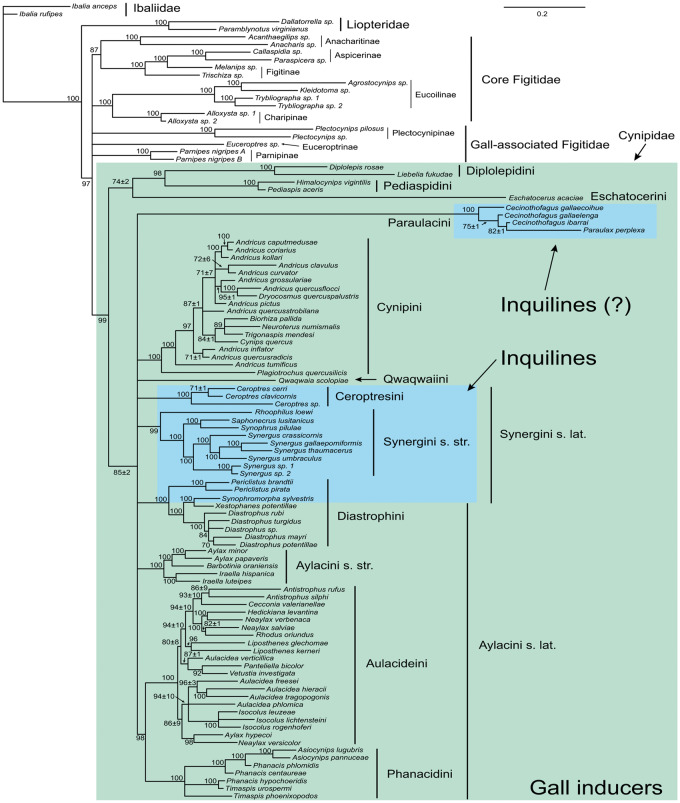
Phylogenetic relationships among cynipids and other gall-associated cynipoids based on a combination of molecular, morphological, and life-history traits (from Ronquist et al. [2015]). Numbers of branches are Bayesian posterior probabilities. Gall inducers and inquilines are marked with different colors.

Initial phylogenetic studies suggested that the ability to induce galls evolved once in the superfamily Cynipoidea, in the ancestor of the Cynipidae. By this hypothesis, though retaining the ability to induce development of nutritive tissues, the cynipid inquilines represented forms that had lost the ability to induce galls de novo (Ronquist 1994, 1995, 1999). This was consistent with the traditional grouping of inquiline genera into a single taxonomic unit, the tribe Synergini sensu lato, which presumably had a single origin from gall inducers. However, more recent studies (Nylander et al. 2004; Ronquist et al. 2015) suggest that the inquilines are polyphyletic and may at least partly represent ancestral rather than derived forms within the family. The latest phylogenetic study splits all the extant Cynipidae into 12 tribes, and inquilines into three tribes: Synergini sensu stricto, Ceroptresini, and Diastrophini, the last of which also includes gall inducers (Ronquist et al. 2015). The relationships are still poorly resolved to some extent, but the available evidence suggests that there have been multiple transitions between gall inducers and inquilines, although the ancestral state is often unclear (Ronquist 1994; Liljeblad 2002; Ronquist et al. 2015). For instance, the proposed monophyletic tribe Diastrophini includes two genera of inquilines and two genera of gall inducers, and the relationships are such that there must have been at least two transitions between inquilines and gall inducers (fig. 1). There could have been two transitions from inquilines to gall inducers, two transitions from gall inducers to inquilines, or one transition in either direction. This makes it quite difficult to use comparative studies in identifying putative adaptations associated with the origin of the ability to induce galls in cynipids.

**Fig. 2.**
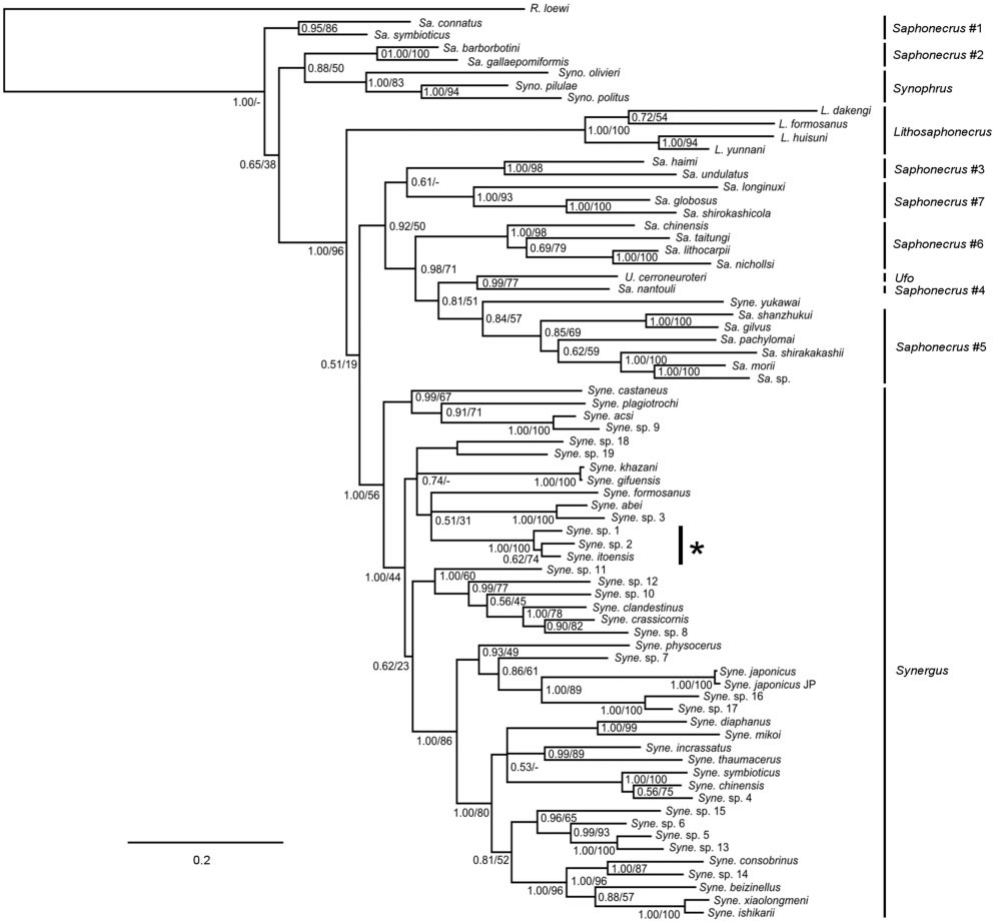
Phylogenetic relationships among species of *Synergus* and related genera in the tribe Synergini sensu stricto based on COI and 28S data, 1.1 kb in total (modified from Ide et al. [2018]). Numbers on branches represent Bayesian posterior probabilities and bootstrap support values from a maximum likelihood analysis. The star indicates species that are known or suspected to be true gall inducers.

The species-rich genus *Synergus* is interesting in this context because it contains one of the few life-history transitions in the Cynipidae that can be reconstructed with some degree of confidence. Until recently, the genus *Synergus* was considered by most to comprise only inquilines. The few apparent exceptions that have been reported in the literature have been contested because they were not properly documented (Ronquist 1994), or have been contradicted by detailed life-history studies (Wiebes-Rijks 1979). However, one *Synergus* species, *Synergus itoensis*, has been shown experimentally to induce galls inside the acorns of its host, *Quercus glauca*, when the acorns are shielded from attack by gall-inducing cynipids (potential hosts) (Abe et al. 2011). Even more interestingly, *S. itoensis* was later shown to be closely related phylogenetically to known inquilines in the genus (Ide et al. 2018), therefore suggesting a relatively recent transition from inquilines to gall inducers in the branch leading to *S. itoensis* (fig. 2). Even though it is possible that the remote ancestors of *S. itoensis* once had the ability to induce galls before becoming inquilines, it appears that the trait must have been restored recently, after a long period of not being expressed. Thus, the origin of the ability to induce galls in the ancestors of *S. itoensis* represents a golden opportunity to use comparative genomics to identify candidate genes involved in the mechanism of gall induction, specifically in the initiation of gall formation.

Here, we apply comparative phylogenomic analyses to *S. itoensis* and three closely related *Synergus* inquiline species to identify putative genomic changes associated with the origin (or restoration) of the ability to induce galls de novo in this lineage. Patterns of synonymous and nonsynonymous substitutions (d*N*/d*S*) were analyzed to identify genes affected by changes in selective pressure. More specifically, we used two alternative approaches based on codon models: 1) a classical fixed-effect branch codon model (implemented in PAML) for detecting episodic changes in selection pressure, applied separately to each gene and 2) a novel Bayesian multigene approach capturing branch–gene interactions in the patterns of d*N*/d*S* across the entire data set. Our multigene approach bears some resemblance with a method previously introduced and applied to placental mammals (Wu et al. 2017) but the specific model and Bayesian Markov chain Monte Carlo inference machinery used here was developed for this study and implemented in a new software. The software is generic and the method can easily be applied to other data sets. A distinct advantage of the Bayesian multigene approach is that it controls for the general level of selection associated with each individual branch in the tree, unlike gene-wise analyses. Branch-specific selection effects may arise, for instance, due to differences in the population-genetic history of different lineages.

## Materials and Methods

### Material

The genomes of four *Synergus* species were used for this study: Previously published genomes of the inquilines *Synergus umbraculus* (Bunnefeld et al. 2018; Hearn 2018) and *Synergus japonicus* (Hearn 2018) and de novo genomes sequenced for this study of the inquiline *S. gifuensis* and the gall-inducer *S. itoensis*. Acorns of *Q. glauca* containing galls of *S. itoensis* were collected in Ito Campus of the University of Kyushu, Fukuoka, Japan (33.5971263°N, 130.2124728°E) on November 21, 2016. Galls of *Andricus kashiwaphilus* were collected on *Quercus dentata* in Jizoubaru, Oita prefecture, Japan (33.1599691°N, 131.1730248°E), on November 22, 2016. Only one *A. kashiwaphilus* gall showed the aberrant morphology associated with the presence of the inquiline *S. gifuensis*. Galls were subsequently dissected in early 2017 and DNA extracted from single larvae using the Thermo Scientific KingFisher Cell and Tissue DNA Kit and the KingFisher Duo magnetic particle processor. The identity of specimens was verified by Sanger sequencing of the COI barcoding fragment and searching the results against the NCBI “nt” database using BLAST (Altschul et al. 1990). The COI sequence of the larvae obtained from the *A. kashiwaphilus* gall matched the barcodes of *S. gifuensis* (GenBank accession number LC272567.1) and of *Synergus khazani* (GenBank accession number KR270557.1), in both cases with 100% identity for the unambiguously called bases in our sequence. The recently described *S. khazani* is very closely related to *S. gifuensis* (Ide et al. 2018), possibly conspecific. Therefore, the specimen will be referred to as *S. gifuensis* in this publication.

### Genome Sequencing, Assembly, and Identification of Orthologs

The two de novo genomes were sequenced from ChromiumX libraries (Zheng et al. 2016) by the Swedish National Genomic Infrastructure using Illumina technology. Clustering was done by “cBot” and samples were sequenced on HiSeqX (HiSeq Control Software HD 3.4.0.38/RTA 2.7.7) with a 2 × 151-bp setup using “HiSeq X SBS” chemistry. The Bcl to FastQ conversion was performed using bcl2fastq-v2.17.1.14 from the CASAVA software suite. The quality scale used was Sanger/phred33/Illumina 1.8+. Filtering and assembly were done by running 10X Genomics’ Supernova version 1.2 using a –bcfrac parameter (and the corresponding –maxreads parameter) from 0.20 to 0.45 in 0.05 steps. The –bcfrac value that gave the most contiguous assembly (0.30 in both cases) was selected for the final assembly.

N50s of the assemblies were 362,131 for *S. itoensis*, 556,258 for *S. gifuensis*, and median contig lengths were 5,711, and 4,824, respectively. Both assemblies had good BUSCO scores: 93% complete and single-copy BUSCOs for *S. itoensis* and 95% for *S. gifuensis* (282 and 287, respectively, out of the 303 eukaryotic groups from the Eukarya_odb set). For the preexisting assemblies of *S. japonicus* and *S. umbraculus*, the N50s were 61,479 and 49,302, and the median contig lengths were 35,864 and 15,571, respectively (see supplementary table S1, Supplementary Material online).

We used Augustus version 3.2.0 (Stanke and Morgenstern 2005) to identify coding sequences in the four genomes, with *Nasonia vitripennis* (the closest available organism phylogenetically) as the training set. We retrieved 23,718 coding sequences for *S. gifuensis*, 23,439 for *S. itoensis*, 19,392 for *S. japonicus*, and 21,814 for *S. umbraculus*. These results are consistent with *S. japonicus* being the least fragmented assembly, and the higher number of coding sequences for the other genomes being due to their assemblies being more fragmented.

Genes from the four species were clustered using Orthofinder version 1.1.8 (Emms and Kelly 2015), resulting in the detection of 15,343 orthogroups, of which 9,955 were represented in all four species. Of the latter, 8,032 were single-copy orthogroups (SCOs), and these were kept for sequence alignment and selection analyses. The percentage of genes that were assigned to an orthogroup ranged between 76.8% and 89.9% in the four species.

### Gene Duplication Analysis

Among Cynipidae, gall inducers have been reported to often have large genome sizes compared with inquilines (Lima 2012). It is unclear whether this is always the case or whether there is a causal relationship. Nevertheless, given the potential of whole-genome duplications as an evolutionary force (MacKintosh and Ferrier 2018), we first investigated the possibility of gene duplication in our *Synergus* data.

To check for major duplication events, we used the single- and double-copy orthologs identified by Orthofinder. For each species, we counted the orthogroups that had exactly twice as many genes as compared with the other three species. We then compared the number of genes falling into this pattern in each species to see if any of the species deviated significantly from the average. The selection analysis could not be readily applied to orthogroups that were not single-copy, so these genes were excluded from further consideration.

### Sequence Alignment

First, we used ClustalOmega version 1.2.4 (Chojnacki et al. 2017) (default parameters) to align the protein sequences of the four orthologs for all the 8,032 SCO genes. Then each protein alignment was filtered with Gblocks version 0.91b (Castresana 2000) to mark unreliably aligned regions, using these parameters: −b1 = 4 −b2 = 4 −b3 = 5. The first two parameters are to ensure that all the blocks have sequences for all four species, since they would not be informative for d*N*/d*S* analysis otherwise. The last parameter is the maximum number of contiguous nonconserved positions and was set at 5 to get a more stringent filtering. To evaluate the effectiveness of the Gblocks filtering strategy, we devised a naïve consensus method and applied it to each alignment. Specifically, we created a 50% consensus sequence using the following rule: For each position of the alignment, if there was a gap in 50% of the sequences, the consensus would have a gap in that position, otherwise it would have the letter that was found in at least 50% of the sequences, or an X if more than one letter was found. We then computed the identity and similarity scores to the consensus for each individual sequence in the alignment, assuming that low identity and similarity scores indicated poor alignments. We plotted the scores against various statistics (ratio between the highest number of gaps and the length of that sequence, proportion of alignment positions without gaps, the negative logarithm of the aforementioned, and the length of the alignment) to see which of them was more effective for identifying poor alignments. The number of positions remaining after Gblocks filtering was the statistic that gave the clearest results: Alignments of length <50 always had low scores, whereas alignments above that length rarely had extremely low scores (supplementary fig. S2, Supplementary Material online). To be on the safe side, a minimum alignment length of 100 positions was judged as an appropriate threshold to remove misaligned sequences. This resulted in a final set of 7,564 protein alignments. Finally, the nucleotide sequences corresponding to the coding sequences of these genes were aligned with PAL2NAL v14 (Suyama et al. 2006) using the protein alignments left after filtering as guide.

The entire procedure described above was then repeated using Muscle v3.8.1551 (Edgar 2004) instead of ClustalOmega to align the protein sequences to add a control for the effect of alignment method, again with default parameters. Of the 8,032 SCO genes, 7,567 had at least 100 positions remaining after Gblocks filtering of the Muscle alignments.

### Species Tree

A species tree was inferred with MrBayes version 3.2.6 (Ronquist et al. 2012) using the sequences of 300 proteins randomly selected from the list of SCOs and aligned with ClustalOmega filtered with Gblocks as described above. The 300 protein alignments were concatenated and analyzed under the mixture of empirical amino acid rate matrices implemented in MrBayes. Specifically, MrBayes settings were left at default values except for the following: aamodelpr = mixed, rates = invgamma, nruns = 4, nchains = 4, ngen = 200,000.

### Selection Analysis Using a Gene-Wise Branch Model

In order to detect changes in selection pressure associated with specific genes and branches in the tree, we first performed an analysis in PAML version 4.9f (Yang 2007). Specifically, each nucleotide alignment was tested with Codeml for six different branch models: No difference in d*N*/d*S* among the four species (null hypothesis, model 0) and higher selection pressure in each one of the five branches of the tree (model 2). Some other relevant options of the control file were as follows: seqtype = 1, CodonFreq = 2, NSsites = 0, fix_kappa = 0, kappa = 2, fix_omega = 0, omega = 2. Genes that had a d*N*/d*S* >3 in at least one of the branches in the branch tests were discarded, as such extreme values are likely artifacts. After the removal of these outliers, we were left with 7,036 genes from the Clustal data set and again 7,036 genes in the case of Muscle, of which 6,987 were present in both analyses.

### Selection Analysis Using an Integrative Branch–Gene Interaction Model

The analysis using a branch model applied to each gene separately is suboptimal in that it does not use all data in a simultaneous analysis to tease apart genome-wide branch effects from effects that are specific to individual gene and branch combinations. Genome-wide branch effects can easily arise because of population-genetic differences among lineages. For instance, a branch with a small effective population size is likely to be associated with more nonsynonymous changes than one with a large effective population size (Ohta 1995; Nikolaev et al. 2007). Controlling for such differences is important in correctly pinpointing the genes that deviate from the expected selection regime because they are associated with the origin of some new trait of interest. In general, we expect genes involved in the origin of a new trait to be positive outliers, that is, associated with less constraining selection than expected, perhaps even positive selection.

To take advantage of the genome-wide signal in the selection analysis, we conducted a global analysis of all genes using an integrative branch–gene interaction model similar in spirit to the approach introduced in Wu et al. (2017). To this end, we developed a new multigene hierarchical phylogenetic codon model, which can be seen as a generalized linear mixed model, expressing the d*N*/d*S* for a given gene *i* over branch *j* as the product of a gene-specific effect, a branch-specific effect, and a residual deviation. The gene effect captures whether a given gene is globally more or less conserved across all species, whereas the branch effect is meant to capture genome-wide variations in the efficiency of purifying selection across species (typically caused by variation in long-term effective population size between lineages). Finally, the deviation from this product captures specific and local changes in the functional conservation/selective constraint for the gene of interest. In particular, a positive deviation suggests that the d*N*/d*S* for gene *i* over branch *j* is elevated, given what we would expect based on the overall level of conservation of that gene, and given the overall strength of selection over that branch. The reason for this deviation could be either that this gene has undergone an episode of positive selection, or that it has been subject to a relaxation of the selective constraint acting on it. Similarly, a negative deviation suggests a local increase in the strength of purifying selection (or, less likely, a decrease in the strength of positive selection).

The model was implemented using BayesCode, a new programming environment for Bayesian inference by MCMC more specifically devoted to exome-wide phylogenetic codon analyses (for more information, see Supplementary Material online). Two independent runs were performed, each using 20 threads for 36 h, resulting in ∼1,000 generations per run. The first 200 generations were discarded as burn-in and the remainder were used to estimate for every branch-gene combination the posterior probability of the branch-gene effect being positive, that is, the posterior probability *P* of the gene having an elevated d*N*/d*S* ratio on that branch compared with the expectation. By construction of the model, the posterior probability *q* of the gene having a lower d*N*/d*S* ratio on the branch than expected (i.e., a negative deviation) is the complement of *P*, that is, *q *=* *1 − *P*. Convergence of parallel chains was assessed by evaluating the correlation coefficient between the selection factors and *P* values estimated by each chain.

### Comparison with Gene Expression Data

A recent transcriptomic study found ∼80 genes to be differentially expressed by larvae of *Biorhiza pallida*, a gall-inducing cynipid, early in gall development relative to later gall developmental stages (Hearn et al. 2019). If these 80 genes include loci associated with induction of a complete gall (and not just nutritive tissues), we might expect their homologs in *Synergus* to include loci showing contrasting signals of selection in the branch leading to *S. itoensis* relative to inquiline *Synergus* species. To address this, the set of protein sequences from *S. itoensis* SCOs was formatted into a database that could be searched for matching sequences of *Biorhiza* using Diamond v.2.

### Gene Set Enrichment Analysis

The protein sets for each species were annotated using eggNOG-mapper v4.5 (Huerta-Cepas et al. 2016). The resulting GO terms were used in a gene set enrichment analysis (GSEA), comparing each candidate set of genes against the full set using topGO v.2.24.0 (Alexa and Rahnenfuhrer 2017).

For all GSEA runs, node size was set to 5, in order to remove GO sets with fewer than this number of gene members, and the method “parentchild” was used with significance determined using Fisher’s exact test. The “parentchild” method considers a given term’s parents in the hierarchy to reduce false-positives arising from inherited relationships (Grossmann et al. 2007). For all species (plus the internal branch), both the Biological Processes and Molecular Function GO categories were assessed, and a conservative significance threshold of 0.001 was used to account for multiple testing, in lieu of a resampling-based approach (Westfall et al. 1993; Grossmann et al. 2007). The procedure was repeated on the outlier sets (the positive signature genes [PSGs] and negative signature genes [NSGs], see below) from all four runs of Bayescode. Additional analyses involved the use of the “weight01” method, also known as the topGO method as it is the default within topGO, which incorporates the GO hierarchy along with the significance scores of connected nodes in the hierarchy, making for a more robust, though conservative test (Alexa et al. 2006).

## Results

### Gene Duplication Analysis

Genes from 141 orthogroups were seemingly present in two copies in *S. itoensis* but only in a single copy in the other three species; this does not necessarily imply a duplication, since genes that are split between two contigs are also included in this count. Other species had similar numbers of putatively duplicated genes: 115 for *S. gifuensis*, 124 for *S. japonicus*, and 94 for *S. umbraculus*. Very few orthogroups that were usually represented by two genes had apparently been duplicated to four in one species (4 in *S. japonicus*, 0 in *S. umbraculus*, and 1 in *S. itoensis* and *S. gifuensis*). Overall, there was no sign of increased duplication activity associated with the origin of the ability to induce galls.

### Alignment and Filtering Results

The SCO alignments obtained with Clustal and Muscle did not differ significantly in average length of selected blocks after Gblocks filtering (1,689 and 1,691 bp, respectively). The number of genes remaining after filtering was also virtually the same (7,564 and 7,567, respectively, with an overlap of 7,552).

### Species Tree

**Fig. 3.**
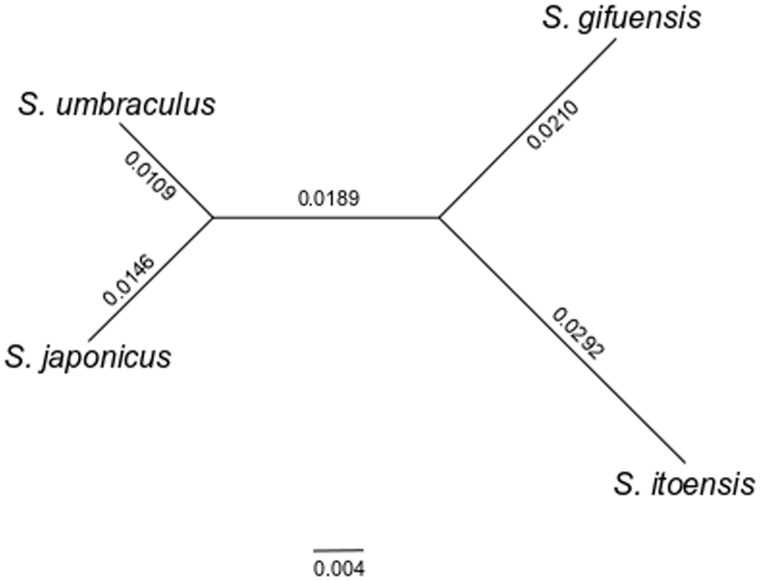
Unrooted tree depicting relationships among the four studied *Synergus* species. The tree was inferred using MrBayes from the protein sequences of 300 randomly selected single-copy genes present in all species. The topology was supported in all sampled trees. Numbers on branches are average branch lengths (expected substitution per amino acid site).

The unrooted species tree inferred from the concatenated protein alignments of 300 randomly selected single-copy orthologs had 100% posterior probability. The tree groups *S. itoensis* and *S. gifuensis* on one side, and *S. japonicus* and *S. umbraculus* on the other (fig. 3). These relationships are consistent with the analysis of Ide et al. (2018), based on partial sequences of cytochrome c oxidase subunit I and 28S ribosomal RNA (1.1 kb of data). However, their analysis only included three of the four *Synergus* species studied here (*S. umbraculus* was not included). Therefore, our tree cannot be rooted using solely the information in Ide et al. (2018), but their analysis indicates that the root in our tree is neither on the terminal branch leading to *S. itoensis*, nor on the branch leading to *S. gifuensis*. This is consistent with our assumption of a recent evolution of gall-inducing ability in the *S. itoensis* branch.

### Selection Analysis Using the Gene-Wise Branch Model

For the gene-wise selection analysis using PAML, we labeled a gene as a “signature gene” for a certain branch if its d*N*/d*S* ratio was significantly higher or lower on that branch than on other branches in the tree (χ^2^ statistic >3.8415 [*P *≤* *0.05] for the alternative hypothesis [model 0] of there being no difference). We term a gene with significantly higher d*N*/d*S* a PSG, and a gene with significantly lower d*N*/d*S* an NSG. Note that these definitions apply to the comparison among branches in the tree, regardless of the average d*N*/d*S* for the gene. In addition, note that, we measure the selection coefficient of the entire gene. Thus, PSGs may not have d*N*/d*S* values >1 even if they are affected by positive selection because such extreme selection regimes may be of short time duration and may not affect more than part of the gene.

The results of this analysis (table 1) indicate that the branch leading to *S. itoensis* does not have a uniquely elevated number of PSGs compared with the branches leading to the other three species. The branches leading to *S. japonicus* and *S. umbraculus* each have about one-third the number of PSGs as the branches leading to *S. itoensis* and *S. gifuensis*, but there is no clear difference between the latter two. NSGs show a different pattern but also fail to indicate that *S. itoensis* is unique: The NSGs are more abundant in *S. japonicus* and less abundant in *S. gifuensis* compared with the other two species. The only way in which *S. itoensis*, the gall inducer, differs from the other species in the gene-wise selection analysis is in its relatively high numbers of both PSGs and NSGs. The significance of the observed patterns is not immediately obvious. However, the analysis using the integrative branch–gene interaction model (see below) indicates that the number of PSGs and NSGs identified on the different branches in the tree is strongly affected by branch-specific genome-wide effects.

**Table 1:** Results from Analysis of the Gene-Wise Branch Model Using Codeml (PAML)

	Positive Signature Genes			Negative Signature Genes		
Species/Branch	Muscle	ClustalOmega	Intersection	Muscle	ClustalOmega	Intersection
*Synergus itoensis*	**183**	**211**	**146**	**169**	**198**	**137**
*Synergus gifuensis*	219	256	180	66	68	50
*Synergus japonicus*	74	91	62	318	324	283
*Synergus umbraculus*	75	92	60	195	208	169
Internal branch	340	398	312	76	83	54

Note.—Positive signature genes (PSGs) and negative signature genes (NSGs) were identified as those genes with a log-likelihood ratio >3.8415 (*P *≤* *0.05) for the hypothesis of a higher d*N*/d*S* in the foreground (PSGs) or in the background (NSGs) subtree. Values for the gall inducer (*S. itoensis*) in bold.

Alignment length and log-likelihood ratio for the hypothesis of two different d*N*/d*S* regimes had a Pearson correlation coefficient of 0.13 for Muscle and 0.14 for Clustal (both above the critical value of 0.03 for significant correlation of ∼7,500 paired data points at 0.01 significance). Thus, longer genes were slightly more likely to give a positive result in the gene-wise selection analyses. The alignment method had a strong impact on the results of the analyses. The correlation coefficient between log-likelihood ratios obtained with different alignment methods for the same gene and branch was only 0.74. Nevertheless, the d*N*/d*S* ratios were fairly consistent between alignment methods (correlation coefficient 0.98 for background, 0.94 for foreground, and 0.92 for delta-d*N*/d*S*).

### Selection Analysis Using the Integrative Branch–Gene Interaction Model

For the branch–gene interaction analysis using the Bayesian multigene approach, we labeled the genes that had a posterior probability >95% of having a significantly elevated d*N*/d*S* for a particular branch as being PSGs. The genes that had a posterior probability <5% of having an elevated d*N*/d*S* for a branch were labeled as NSGs; by construction of the model, those genes also had a posterior probability >95% of having a lower d*N*/d*S* than expected. According to these definitions, the branch leading to *S. itoensis* has distinctly more PSGs and NSGs than any of the other branches in the tree (tables 2 and 3).

**Table 2:** Positive Signature Genes (PSGs) Identified in the Integrative Gene–Branch Interaction Model Using Bayescode

	Muscle			ClustalOmega			
Species/Branch	Run 1	Run 2	Both Runs	Run 3	Run 4	Both Runs	All Runs
*Synergus itoensis*	**190**	**197**	**183**	**228**	**234**	**218**	**145**
*Synergus gifuensis*	154	155	148	183	185	177	108
*Synergus japonicus*	159	155	150	189	196	182	121
*Synergus umbraculus*	142	142	138	161	164	160	110
Internal branch	119	122	114	130	130	120	100

Note.—PSGs were identified as those genes with posterior probability ≥ 0.95 for the hypothesis of the gene having a d*N*/d*S* ratio higher than expected from the interaction between the gene factor and the branch factor. The gall inducer *S. itoensis* consistently displays the highest number of PSGs (values in bold).

**Table 3:** Negative Signature Genes (NSGs) Identified in the Integrative Gene–Branch Interaction Model Using Bayescode

	Muscle			ClustalOmega			
Species/Branch	Run 1	Run 2	Both Runs	Run 3	Run 4	Both Runs	All Runs
*Synergus itoensis*	**234**	**226**	**210**	**274**	**277**	**256**	**167**
*Synergus gifuensis*	133	129	119	148	150	138	90
*Synergus japonicus*	63	60	54	82	83	75	44
*Synergus umbraculus*	54	49	45	55	60	48	36
Internal branch	157	159	146	168	179	153	102

Note.—The NSGs were identified as those genes with posterior probability ≥ 0.95 for the hypothesis of the gene having a d*N*/d*S* ratio lower than expected from the interaction between the gene factor and the branch factor. The gall inducer *S. itoensis* consistently displays the highest number of NSGs (values in bold).

The estimated genome-wide branch effects seem to have a distinct effect on the number of PSGs and NSGs detected by PAML (table 1). The branches with the lowest d*N*/d*S* ratios across the genome (*S. japonicus* and *S. umbraculus*; estimated branch effects of 0.60–0.66) are also the branches where PAML finds a small number of PSGs and a large number of NSGs. Conversely, the branches with the highest d*N*/d*S* ratios (*S. gifuensis* and the internal branch; estimated branch effects of 1.0–1.1) are the branches where PAML detects a large number of PSGs and a small number of NSGs. Thus, the fact that the gene-wise analysis with PAML does not control for the genome-wide branch effects introduces a potential bias, making it more difficult to identify the genes with deviating d*N*/d*S* ratios. There is no such imbalance between PSGs and NSGs in the results from the integrative model (tables 2 and 3).

**Fig. 4.**
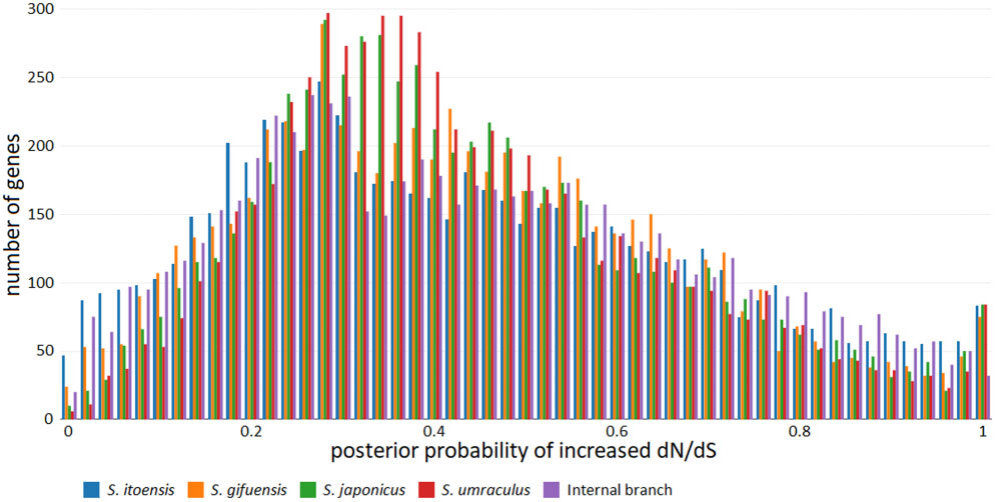
Distribution of the Bayescode posterior probabilities, *P*, of the gene effects in the gene–branch interaction model being positive in each of the five branches. Data from run 2, based on the Muscle alignment. The posterior probability of the gene effect being negative, *q*, is the complement of *P*, that is *q *=* *1 − *P*. If *P* is very high, say >95%, then the gene is likely to have had an elevated d*N*/d*S* on the selected branch compared with what would have been expected for that gene and that branch. Conversely, if *P* is very low (and *q* high), the gene is very likely to have had a lowered d*N*/d*S* on the selected branch.

The distribution of the posterior probabilities of a branch-gene effect being positive varies between branches (fig. 4). There is a small but distinct peak at posterior probabilities close to 100%; this could be due in part to alignment errors or other artifacts. Such a peak is absent at the other end of the scale, at posterior probabilities close to 0%. The posterior probabilities are clearly more spread out for the *S. itoensis* branch than for the three branches leading to inquiline species, even if the supposedly artifactual peak close to 100% is disregarded. The interior branch also has a tendency toward overrepresentation of low and high posterior probabilities, but not quite to the same extent as the branch leading to *S. itoensis* (see also tables 2 and 3).

Parallel runs returned highly concordant results (posterior probabilities and branch-gene effects having a correlation of 0.99 for both Clustal and Muscle aligned data sets). There was virtually no correlation between alignment length and the posterior probability of having an elevated d*N*/d*S* ratio for a particular branch (Pearson correlation coefficient around −0.015 with both alignment methods, *P *>* *0.01). Results from the branch–gene interaction analysis were not affected significantly by the alignment method: The correlation coefficients between the two alignment protocols were 0.94 for posterior probabilities and 0.93 for branch-gene effect coefficients.

### Comparison of the Gene-Wise and Integrative Model

Results from the gene-wise branch model analysis and the integrative branch–gene interaction analysis were not strongly correlated: Genes with a low log-likelihood ratio in the gene-wise analysis were distributed throughout the entire range of posterior probabilities in the integrative analysis (supplementary figs. S2 and S3, Supplementary Material online). However, genes with higher log-likelihood scores tended to cluster toward either the low or the high end of the posterior probability range, as expected. The Spearman’s rho between the two scores varied a lot between branches and averaged ∼0.72 for Muscle alignments and 0.73 for Clustal alignments.

We also compared the PSGs and NSGs identified by Bayescode with those that had a log-likelihood score >3.84 (*P *<* *0.05) in the PAML analysis (supplementary table S2, Supplementary Material online). Averaged across species, only 60% of PSGs and 75% of NSGs identified by Bayescode were also identified by PAML. Because of the more elaborate model structure, and the higher consistency of the results of the integrative analyses across alignment methods, we focused on the latter in the concluding tests.

### Comparison with Expression Data from *B. pallida*

We found no overlap between the sets of PSGs and NSGs identified in our study and the genes that are overexpressed or underexpressed during early stages of gall induction in the oak gall wasp *B. pallida* (Hearn et al. 2019).

### Automatic Annotation of Genes Using eggNOG-mapper

Of the 8,032 protein sequences of *S. itoensis*, 7,576 were matched to an ortholog in the eggNOG database with a bit-score >60 and an e-value <1.0e-5. This amounted to 96.0% of either of the sets of alignments that were used in the d*N*/d*S* analysis. Among Bayescode’s PSGs and NSGs, 94.9% and 98.9% could be annotated.

### Gene Set Enrichment Analysis

GSEA using the Molecular Function GO annotations found no significantly enriched categories among the PSG and NSG sets identified by the integrative model. However, analysis of Biological Processes annotations returned several categories with low and significant *P* values (*P *<* *0.001) in the PSGs of the gall-inducing *S. itoensis* (table 4). In the PSGs for all other branches, the GO terms with the lowest *P* values were always above this threshold (except for two terms that showed up in no more than two of the four Bayescode runs and are probably false-positives). Four terms seem to be enriched on the internal branch in all four runs, although their *P* values are above the threshold (“imaginal disc lineage restriction,” “regulation of protein stability,” “process utilizing autophagic mechanism,” and “negative regulation of cell cycle process”). No enriched categories were found among the NSGs.

**Table 4:** Results from the Gene Set Enrichment Analysis (GSEA) of Gene Ontology (GO) Terms

GO ID	GO term	Muscle Alignment		Clustal Alignment		Nonspecific Occurrences	
		Run 1	Run 2	Run 3	Run 4	Number of Occurrences	Lowest *P* Value
GO:0030707	Ovarian follicle cell development	0.00015	0.00051	0.00292	4.2e-05	—	
GO:0007507	Heart development	0.00025	0.0021	0.00049	0.00049	2	0.0227
GO:0007409	Axonogenesis	0.00043	0.0082	7.8e-05	0.00066	—	
GO:0061564	Axon development	0.00097	0.01536	0.00021	0.00166	—	
GO:0042221	Response to chemical	0.00099	0.00171	0.03052	—	—	
GO:0007320	Insemination	—	—	0.00075	0.02168	—	
GO:0006928	Movement of cell or subcellular component	0.00227	0.00305	0.00078	9.4e-05	—	
GO:0007498	Mesoderm development	—	—	0.00431	0.00035	—	0.02539
GO:0040011	Locomotion	0.00768	0.00858	0.0037	0.00054	3	0.02232

Note.—The *P* value of the parent–child Fisher’s test is given for all the Bayescode runs in which a term is overrepresented among the PSGs of *Synergus itoensis*. When the term is overrepresented also in a branch other than *S. itoensis*, the number of nonspecific occurrences is given together with the lowest *P* value among those runs.

The GO terms that were significantly enriched among the PSGs of *S. itoensis* in at least one run are listed in table 4. Four terms were consistently and significantly enriched in *S. itoensis*: “ovarian follicle cell development,” GO:0030707; “heart development,” GO:0007507, “axonogenesis,” GO:0007409; and “axon development,” GO:0061564.

As an example of the GSEA results, the hierarchical subgraph of the top five GO terms for *S. itoensis* in Bayescode (run 1) is shown in supplementary figure S4, Supplementary Material online.

## Discussion

Our results indicate that duplication of a significant part of the genome is not involved in the acquisition of the ability to induce galls in *S. itoensis*. Some of the duplicated genes we found may nevertheless have a role in gall induction, so further analyses of these genes would probably be worthwhile even though it will require meticulous orthology analysis to minimize the rate of false-positives.

Our selection analysis of SCOs used the standard gene-wise selection analysis, but also a novel Bayesian approach based on an integrative analysis of the interaction between gene effects, branch effects, and gene-branch effects in the entire data set. Clearly, the integrative approach, first proposed by Wu et al. (2017) and implemented independently by us, is preferable from a modeling perspective. Branch-specific differences in selection pressure are expected due to the inherent variation across lineages in population history. Gene-wise analyses are unable to control for such branch-specific effects (except in post hoc analyses) because the effects only emerge if a large set of genes is considered. Thus, we expect gene-wise analyses to find outliers too easily on branches with a generally high selection pressure, and to miss important outliers on branches with a generally low selection pressure. This is exactly the pattern we see when we compare the gene-wise results (table 1) with the estimated genome-wide branch effects from the integrative analysis. This extra noise is reduced in the integrative method by accounting for the expected selection level associated with each branch in the tree, inferred from all genes in the data set. Thus, the integrative selection analysis should be able to pinpoint the genes of interest more precisely than the classical gene-wise analysis. Note the lack of imbalance between PSGs and NSGs across branches in the tree in the integrative analysis (tables 2 and 3), which is consistent with this expectation.

We think that our results support the conclusion that the results from the integrative analysis are indeed more accurate. It is true that the exact composition of the PSG sets and NSG sets is quite sensitive to alignment method and to approximately the same extent for the gene-wise and the integrative analysis. However, the posterior probabilities of the integrative analysis were considerably more robust to the alignment protocol than the likelihood scores from the gene-wise analysis. This occurred despite the fact that we removed some obvious outliers from the gene-wise analysis before measuring the correlation of likelihood scores between alignments, and made no such adjustment for the integrative analysis. The results of the integrative analysis were also essentially independent of alignment length, which was not the case for the gene-wise analysis.

Although we did not remove any extreme outliers in the integrative analysis, we did note an extra peak close to 100% posterior probability of having a positive branch-gene effect in all the terminal branches of the tree (fig. 4). Interestingly, this extra peak is absent from the spectrum of the interior branch, and it is also absent from the low end of the spectrum (posterior probabilities close to 0% of having a positive branch-gene effect) for all branches. One possible explanation is that the extra peak represents artifacts due to alignment errors; their absence from the interior branch could be due to the fact that alignment errors are typically specific to one of the aligned sequences and, as a result, tend to induce artefactual substitution events along terminal branches only.

Given that there are several reasons to consider the integrative analysis more reliable than the gene-wise analysis, it is interesting that it also identifies an elevated rate of genomic change (elevated numbers of PSGs and NSGs) in the branch leading to *S. itoensis*, the branch where apparently the ability to induce galls originated (or was restored if true gall induction was in the ancestry of *Synergus*). There is a tendency for the gene-wise PAML analysis to also associate the branch leading to *S. itoensis* with many PSGs and NSGs, but not to the extent that it stands out from the other terminal branches in the tree. The PSG and NSG sets are also quite different between the gene-wise and integrative analyses for some species, especially for *S. itoensis* (supplementary table S2, Supplementary Material online). Thus, the integrative approach appears to be key to identifying the genomic changes associated with the origin of gall induction in *S. itoensis*.

The selection analysis can pinpoint genes for which the origin of the ability to induce galls is associated with elevated (PSGs) or depressed (NSGs) selection pressure compared with expectation. However, there is only a limited amount of information in this fact alone. First, this apparent association might be coincidental. The genes identified in this way need not necessarily be related to gall induction, and could be associated with any other phenotypic innovation in *S. itoensis* relative to other *Synergus* species, or even to local adaptation in the population that was sampled. Even if the association is not coincidental, increased selection pressure compared with what is expected for the gene and branch may be due, for instance, to positive selection on (some parts of) a gene adapting to a new condition or relaxation of purifying selection in a gene that became (partially) redundant because of the new condition. It is reasonable to speculate that the PSG sets include both of these categories. Similarly, NSG sets are likely to include genes experiencing stronger purifying selection, and potentially also some genes experiencing relaxation of otherwise consistent positive selection (on parts of the gene). Unfortunately, we were not able to find a reliable way of separating these different categories of PSGs and NSGs.

Gene set enrichment analysis is one way of further exploring whether the PSG and NSG sets identified for the *S. itoensis* branch might be involved in gall induction. In particular, if the PSG set of the *S. itoensis* branch includes genes associated with the origin of the ability to induce galls, we may expect the set to be enriched in genes that are related functionally to each other. Indeed, this is what we find. The fact that only *S. itoensis* has enriched gene ontology categories among its PSGs may well reflect the fact that these genes are involved in biological processes that are crucial for the life-history transition from inquilines to gall inducers. Linking the “heart development” category and gall induction appears difficult, but the other three biological process (“ovarian follicle cell development,” “axonogenesis,” and “axon development”) categories that are enriched in *S. itoensis* could potentially have something to do with the gall initiation process, as explained below.

Cynipid eggs have been observed penetrating the plant tissue just after oviposition, which is suggested to be due to enzymatic digestion of the cell walls (Shorthouse et al. 2005). It is quite possible that this process also involves a key step in gall induction, which may be controlled by genes involved in the process of “ovarian follicle cell development.” Follicle cells are responsible for the making of the egg-shell (Dobens and Raftery 2000), which is known to induce plant responses (Hilker and Fatouros 2015). The complete list of PSGs linked to the egg follicle development is given in supplementary table S3, Supplementary Material online. Differences in the development of the nervous system, linked to differences in the process controlled by genes in the “axonogenesis” and “axon development” categories, could potentially be linked to different oviposition behaviors. This is relevant because inquilines always oviposit into gall tissues, and gall inducers target normal plant tissue.

The ability to induce galls in *S. itoensis* was suggested to have evolved independently from other gall wasps, or restored after a long period of not being used (Ide et al. 2018). Thus, it is interesting to ask whether the same genes might be involved in gall initiation in *S. itoensis* and in other cynipids. The lack of overlap between our PSGs and the genes that are overexpressed in the early *B. pallida* larva suggests that this is not the case. However, it is also possible that the critical gall induction genes are overexpressed already in the ovipositing female or in the egg, and may not be detectable in differential expression analyses of young larvae. It is also possible that the critical genes are regulatory, and that they may not be detectable in gene expression analyses. Thus, it is too early to completely dismiss the hypothesis that similar genes are involved.

### Conclusion

Cynipids, with their mastery of gall induction, have long fascinated biologists. How they manipulate plant morphogenesis is a mystery that has proven hard to solve using experimental studies. We think our results demonstrate that comparative phylogenomic studies can serve as an important complementary tool to experimental studies, and can help identify candidate genes involved in the gall induction process. This can, in turn, inspire targeted experimental studies, confirming or rejecting the hypothesis that said genes are linked to gall induction.

Our results only represent the first steps toward identifying genes involved in gall induction in *S. itoensis*. Some reasonable next steps may be transcriptome or expression analysis targeting young galls of *S. itoensis* to examine whether candidate genes are expressed in the critical life-history stages. It may also be interesting to collection population-level data to further characterize selection pressure on different genes in *S. itoensis*, to identify more recent changes that a phylogenetic approach would likely miss, and to map candidate gall-induction genes to physical locations in the *S. itoensis* genome. Just adding more inquiline species, and putative gall inducers related to *S. itoensis*, may considerably improve the accuracy of the selection analysis presented here. Looking at more transitions from inquilines to gall inducers among other cynipids may also be informative.

*Synergus itoensis* apparently evolved its ability to induce galls relatively recently, and its galls are as simple as cynipid galls get: An unremarkable chamber around the growing larva inside the acorn. Clearly, phylogenomic studies targeting *S. itoensis* and its close inquiline relatives can only identify candidate genes that could potentially be involved in initiating and forming such simple galls. Are those the same genes involved in the origin of the ability to induce galls in other cynipids? This is still an open question, but one that is now open to investigation. If there are such links across independent origins of gall-inducing cynipids, this could both strengthen the association between candidate genes and gall induction, and help pinpoint some of the branches in the cynipid tree where gall inducers evolved.

An interesting question is whether the genes involved in gall initiation are the same as those involved in controlling the species-specific traits of the more sophisticated cynipid galls; one might suspect that these are different sets of genes. If so, the gall initiation genes should be associated with strong purifying selection in large clades of gall-inducing cynipids, whereas the gall differentiation genes should be under strong positive selection due to the arms race with the natural enemies of cynipids (Bailey et al. 2009). Comparative phylogenomic studies of, say, large sets of oak gall wasp species should be able to address these questions. Clearly, comparative phylogenomic studies have the potential to significantly advance our understanding of the genetic basis of gall induction in cynipids.

## Supplementary Material

Supplementary data are available at *Genome Biology and Evolution* online.

## Supplementary Material

evaa204_Supplementary_Data
