# Supplementary material for: From Inquilines to Gall Inducers: Genomic Signature of a Life-Style Transition in Synergus Gall Wasps
Source: Genome Biol Evol. 2020 Sep 28;12(11):2060–73. doi: 10.1093/gbe/evaa204 (PMC7674688; doi:10.1093/gbe/evaa204)
Supplement: evaa204_Supplementary_Data [file evaa204_supplementary_data.docx]

Supplementary Material for “From Inquilines to Gall Inducers: Genomic Signature of a Life-style Transition in *Synergus* Gall Wasps”

Erik Gobbo^[[1]](#footnote-2)^*, Nicolas Lartillot^[[2]](#footnote-3)^, Jack Hearn^[[3]](#footnote-4)^, Graham N. Stone^[[4]](#footnote-5)^, Yoshihisa Abe^[[5]](#footnote-6)^, Christopher W. Wheat^[[6]](#footnote-7)^, Tatsuya Ide^[[7]](#footnote-8)^, Fredrik Ronquist1

# Contents

- Branch-gene interaction model implemented in Bayescode
- Table S1. Quality statistics of the four assemblies used in this study.
- Table S2. Number of genes identified as PSGs and NSGs by PAML, Bayescode (run 2), and both of them.
- Table S3. *Drosophila melanogaster* orthologues of the PSGs associated with the GO term “egg-follicle cell development”, and reference to evidence for their biological function
- Figure S1: Alignment quality as a function of alignment length after Gblocks filtering.
- Figure S2: Scatterplot showing the correlation between PAML and Bayescode results when PAML is used to test for genes with dN/dS higher in the foreground than in the background.
- Figure S3: Scatterplot showing the correlation between PAML and Bayescode results when PAML is used to test for genes with dN/dS lower in the foreground than in the background.
- Figure S4: Subgraph of the top 5 GO terms identified in the gene set enrichment analysis for *S. itoensis*.
- References for Table S3

# Branch-gene interaction model implemented in Bayescode

Consider a series of N orthologous groups (hereafter called genes), for P taxa. All genes are assumed to share the same phylogeny, with gene-specific branch lengths. They are assumed to evolve under a Muse and Gaut codon substitution process (Muse & Gaut 1994), parameterized as in Lartillot and Poujol (2011). Generically, this codon substitution process combines a general time-reversible nucleotide substitution process, defined by a 4x4 rate matrix *R*, with a scalar parameter (*omega*), acting multiplicatively on non-synonymous substitution rates. The matrix *R* itself is parameterized in terms of a vector *rho* of 6 exchangeability parameters (constrained to sum to 1), and a vector *pi* of equilibrium frequencies (also summing to 1). Thus, for nucleotides *m* and *n*,

*R_mn_ = 1/Z · ρ_mn_ · π_n_*

where we use the convention that *ρ_mn_ = ρ_nm_* (to enforce time-reversibility) and where *Z* is a normalization factor ensuring that the expected number of nucleotide substitutions per unit of time is equal to 1. From there, the 61x61 codon process is defined as follows. For codons *a* and *b*, differing at one single nucleotide position, with nucleotides m and n at that position:

Q_ab_ = R_mn_ if (a,b) are synonymous

Q_ab_ = R_mn_ · ω if (a,b) are non-synonymous

For any two codons a and b that are not nearest neighbors, the substitution rate is equal to 0:

Q_ab_ = 0 if (a,b) are not nearest-neighbors

This generic codon process is then modulated across genes and branches as follows.

First, the nucleotide parameters are gene-specific. Specifically, for gene *i*, two parameter vectors are invoked ρ*_i_* and π*_i_* of dimension 6 and 4, respectively. On the other hand, a reasonable expectation is that nucleotide rates should nevertheless tend to be similar across genes. Accordingly, soft shrinkage is implemented over the distribution of the *ρ_i_* and the *π_i_* across genes, by assuming that these two gene-specific parameter vectors are independent and identically distributed (i.i.d.) from two hyper-parameterized Dirichlet distributions:

*ρ_i_ ~ Dirichlet ( ρ^0^ · ζ^-1^ ), for i=1..N*

*π_i_ ~ Dirichlet ( π^0^ · ͱ^-1^ ), for i=1..N*

Here, *ρ^0^* and *π^0^* are positive vectors of dimension 6 and 4, both summing to 1. These two vectors tune the center (the mean) of the two Dirichlet distributions. As for *zeta* and *eta*, they are positive parameters, tuning the width of the distributions. The mean parameters, *ρ^0^* and *π^0^* are endowed with a uniform prior over the 6- and 4-simplex, while zeta and eta are endowed with an exponential prior of mean 1. In effect, this hierarchical setting will automatically adjust, through *ρ^0^*, *π^0^*, *ͱ* and *ζ*, both the center and the strength of the shrinkage across genes. In particular, if the data are such that genes have very similar nucleotide frequencies, then *eta* will be inferred to be small, thus resulting in a more focused posterior distribution across genes.

A similar soft-shrinkage device is invoked for branch lengths. Specifically, calling *l_ij_* the length of branch *j* for gene *i*, the model assumes that, across genes, for a given branch *j*, branch lengths are gamma distributed, with mean *l^0^_j_* and shape parameter κ *^-1^*:

*l_ij_ ~ Γ ( l^0^_j_ , κ ^-1^ )*

With this parametrization, for a given branch *j* of the phylogeny, the branch lengths are allowed to fluctuate across genes, around a branch-specific mean of *l^0^_j_* , and with a dispersion that is tuned by the hyperparameter *κ*. Parameterizing the dispersion in terms of the inverse of the shape parameter is more convenient in a soft-shrinkage context: letting *κ* go to zero implements strong shrinkage, essentially imposing the same branch lengths across genes. The branch-specific means are iid from an exponential distribution of mean λ, itself endowed with an exponential prior of mean 0.1. As for *κ*, it is endowed with an exponential prior of mean 1.

Finally, the models allows for variation in dN/dS across genes and branches. Specifically, letting ω*_ij_* denote the dN/dS over branch j for gene i, the model assumes that *omega_ij* is distributed as:

(1) ω*_ij_* ~ Γ (mean = w_i_ · v_j_ , shape = ɑ^-1^),

where w_i_ is a gene-specific effect and v_j_ a branch-specific effect. Equation (1) can be equivalently re-written as follows:

(2) log ω*_ij_* = a_i_ + b_j_ + e_ij_

where a_i_ = log w_i_ and b_j_ = log v_j_ . This reformulation suggests that the model can be seen as a generalized linear mixed model over the branch- and gene-specific modulations of the strength of selection. Thus, a_i_ captures whether a given gene is globally more conserved, across all species, while b_j_ captures genome-wide modulations in the efficiency of purifying selection across species (typically caused by variation in effective population size between lineages). Finally, e_ij_ is the specific deviation for gene i over branch j. A positive e_ij_ , for instance, suggests that the dN/dS for gene i over branch j is too high, given what we would expect based on the overall level of conservation of that gene, and given the overall strength of selection over that branch. In order to implement efficient control over the shrinkage across genes and branches, the gene- and branch-specific effects are themselves drawn from the following hierarchical prior:

v_j_ ~ iid Γ across branches of mean β and shape parameter γ^-1^

w_i_ ~ iid Γ across genes, of mean δ and share parameter ε^-1^

In turn, the hyperparameters of these two priors (as well as the inverse shape parameter alpha) are estimated globally across the entire set of N multiple sequence alignments, under the following hyper-priors: exponential distribution of mean 1 on ɑ, β, γ, δ, ε. In the practical applications considered here, all of these parameters take values of the order of 1 or below, so an exponential of mean 1 can be considered as a sufficiently vague prior.

Of note, and as in the case of nucleotide rates and branch lengths, the use of inverse shape parameters (ɑ, γ, ε) allows for efficient shrinkage in low-variance settings. In particular, letting ɑ -> 0 gives a distribution dN/dS over branches and genes entirely determined by the product of gene- and branch-effects.

The model was implemented in C++, using MPI parallelization so as to analyse all genes in parallel. The code is available at github.com/bayesiancook/bayescode.git. Typically, the model can be run with an average of 100 genes per core, over 24 hours, yielding around 1000 generations.

**Table S1**. Quality statistics of the four assemblies used in this study.

| **Species** | **NG50** | **Median contig length** | **Coding sequences** | **BUSCO (303 gene groups searched)** | | | |
| --- | --- | --- | --- | --- | --- | --- | --- |
|  |  |  |  | **Complete** | **Complete single-copy** | **Fragmented** | **Missing** |
| *S. itoensis* | 362,131 | 5,711 | 23,439 | 287 | 282 | 8 | 8 |
| *S. gifuensis* | 556,258 | 4,824 | 23,718 | 294 | 287 | 5 | 4 |
| *S. japonicus* | 61,479 | 35,864 | 19,392 | 298 | 289 | 1 | 4 |
| *S. umbraculus* | 49,302 | 15,571 | 21,814 | 297 | 288 | 2 | 4 |

**Table S2**: Number of genes identified as PSGs and NSGs by PAML, Bayescode (run 2), and both of them. We used a cut-off of 5% expected false positive rate. Genes with off-scale values of log-likelihood scores are not included in this analysis; hence, the Bayescode PSG sets are slightly smaller than those presented before.

|  | **PSGs** | | | **NSGs** | | |
| --- | --- | --- | --- | --- | --- | --- |
|  | **PAML** | **Bayescode** | **Intersection** | **PAML** | **Bayescode** | **Intersection** |
| *S. itoensis* | 183 | 181 | 121 | 169 | 224 | 134 |
| *S. gifuensis* | 219 | 145 | 107 | 66 | 129 | 56 |
| *S. japonicus* | 74 | 146 | 66 | 318 | 62 | 61 |
| *S. umbraculus* | 75 | 128 | 68 | 195 | 53 | 53 |

**Table S3**: *Drosophila melanogaster* orthologues of the PSGs associated with the GO term “egg-follicle cell development”, and reference to evidence for their biological function

| **FlyBase ID** | **Gene name** | **Description** | **Reference** |
| --- | --- | --- | --- |
| FBgn0000180 | bib (big brain) | Aquaporin | (Dobens & Raftery 2000) |
| FBgn0000146 | aub (aubergine) | piRNA binding protein, translation suppressor | (Klattenhoff et al. 2007) |
| FBgn0015778 | rin (rasputin) | mRNA binding, translation regulator | (Costa et al. 2013) |
| FBgn0000463 | Dl (Delta) | Notch signalling mediator | (Torres et al. 2003) |
| FBgn0002914 | Myb (Myb oncogene-like) | DNA binding, transcription activator | (Beall et al. 2002) |
| FBgn0264695 | Mhc (Myosin heavy chain) | Cytoskeletal protein enabling cell migration | (Borghese et al. 2006) |
| FBgn0003444 | smo (smoothened) | G protein-coupled receptor that transduces the hedgehog signal | (Kirilly et al. 2005) |
| FBgn0000382 | csw (corkscrew) | Required in all receptor tyrosine kinase signalling pathways | (Perkins et al. 1996) |
| FBgn0086655 | jing | Zinc finger protein, probably transcription repressor | (Liu & Montell 2001) |
| FBgn0267390 | dop (drop out) | Kinase required for follicle cell migration | (Aranjuez et al. 2012) |
| FBgn0000182 | BicC (Bicaudal C) | RNA-binding protein required for targeting of follicle cells migration | (Schüpbach & Wieschaus 1991) |
| FBgn0032006 | Pvr (PDGF- and VEGF-receptor related) | Receptor protein-tyrosine kinase involved in follicle cell migration | (Fernández-Espartero et al. 2013) |
| FBgn0003334 | Scm (Sex comb on midleg) | Polycomb group (PcG) protein that maintains transcription repression of homeotic genes | (Narbonne et al. 2004) |
| FBgn0016917 | Stat92E | Involved in signal transduction and activation of transcription | (Jang et al. 2009) |
| FBgn0011655 | Med (Medea) | DNA-binding transcription regulator | (Kirilly et al. 2005) |
| FBgn0003391 | Shg (shotgun) | Cadherin (calcium-dependent cell adhesion protein) | (Cai et al. 2014) |
| FBgn0014380 | RhoL (Rho-like) | Ras-like GTP-binding protein | (Dobens & Raftery 2000) |


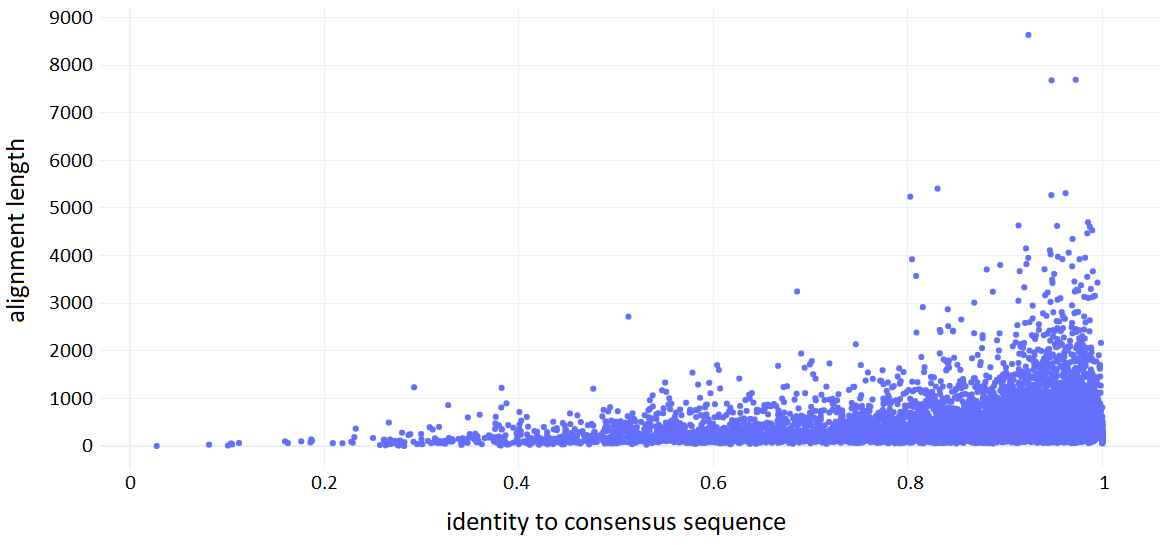


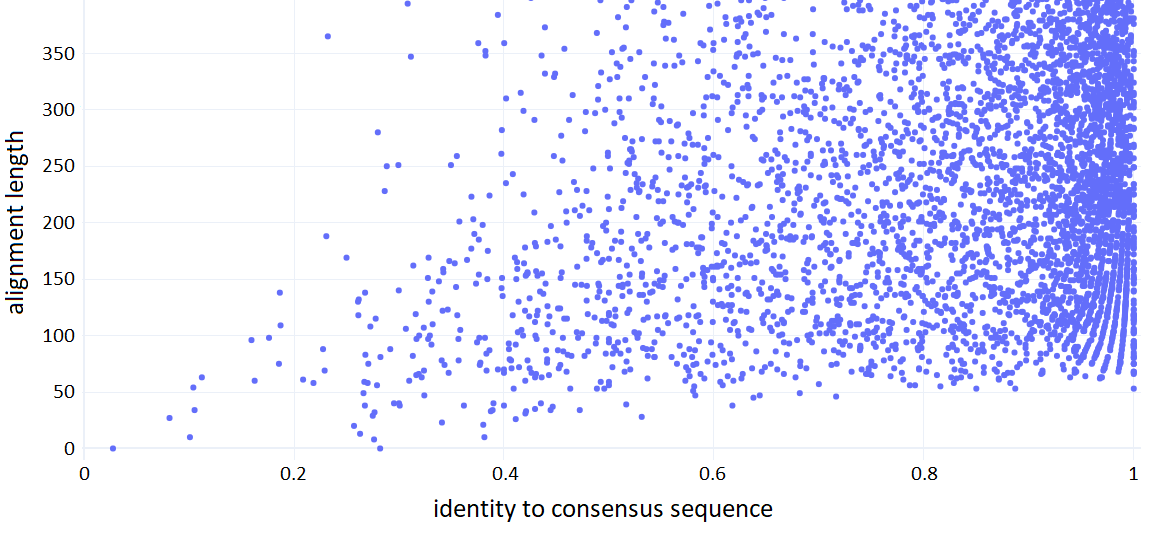


**Figure S1**: Alignment quality as a function of alignment length after Gblocks filtering. Alignment quality was measured as the lowest identity of a sequence with the consensus sequence. Since these are amino-acid sequences, an identity of 20% is extremely unlikely to be the result of chance. Top: The graph shows that sequences with more than 500 amino-acid sites are all of good quality. Bottom: Detail of the lower part of the graph. Alignments with less than 50 sites selected by Gblocks never give a score above 0.75, and most of the alignments with a score below 0.2 have less than 100 sites. A minimum alignment length of 100 was chosen to filter out poor alignments before the selection analysis was applied.


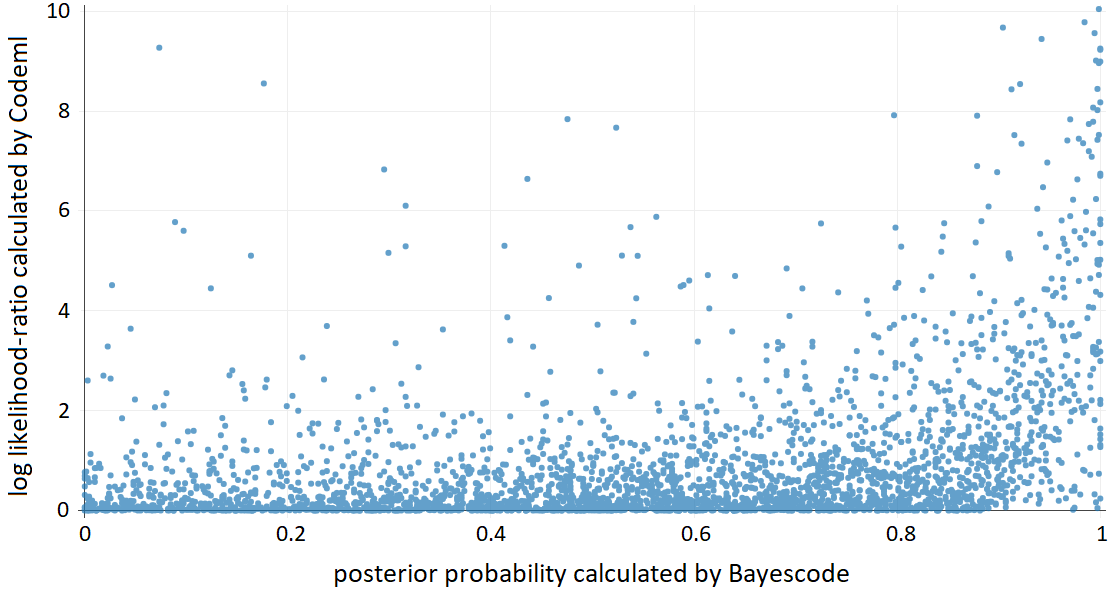


**Figure S2**: Scatterplot showing the correlation between PAML and Bayescode results when PAML is used to test for genes with dN/dS higher in the foreground than in the background. The *y* axis shows the likelihood score from the gene-wise branch model analysis with PAML; the *x* axis shows the posterior probability of a branch-gene effect being positive in the integrative branch-gene model analysis with Bayescode.


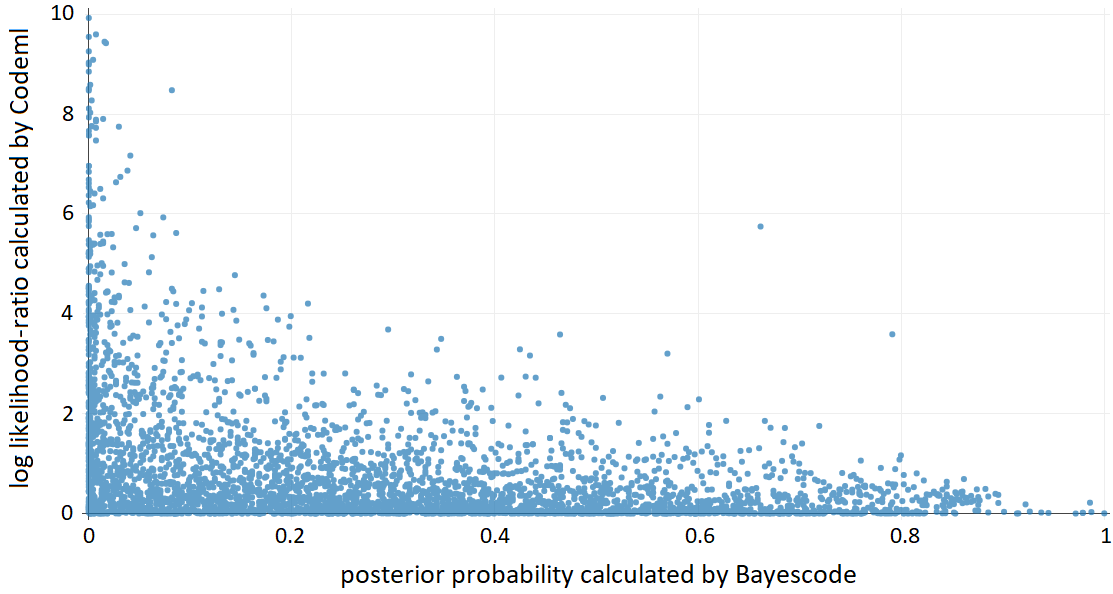


**Figure S3:** Scatterplot showing the correlation between PAML and Bayescode results when PAML is used to test for genes with dN/dS lower in the foreground than in the background. The *y* axis shows the likelihood score from the gene-wise branch model analysis with PAML; the *x* axis shows the posterior probability of a branch-gene effect being positive in the integrative branch-gene model analysis with Bayescode. The posterior probability *p* of a branch-gene effect being negative is the complement of the probability *q* of it being positive, that is, *p* + *q* = 1. Note that it is *q* that is shown on the *x* axis.


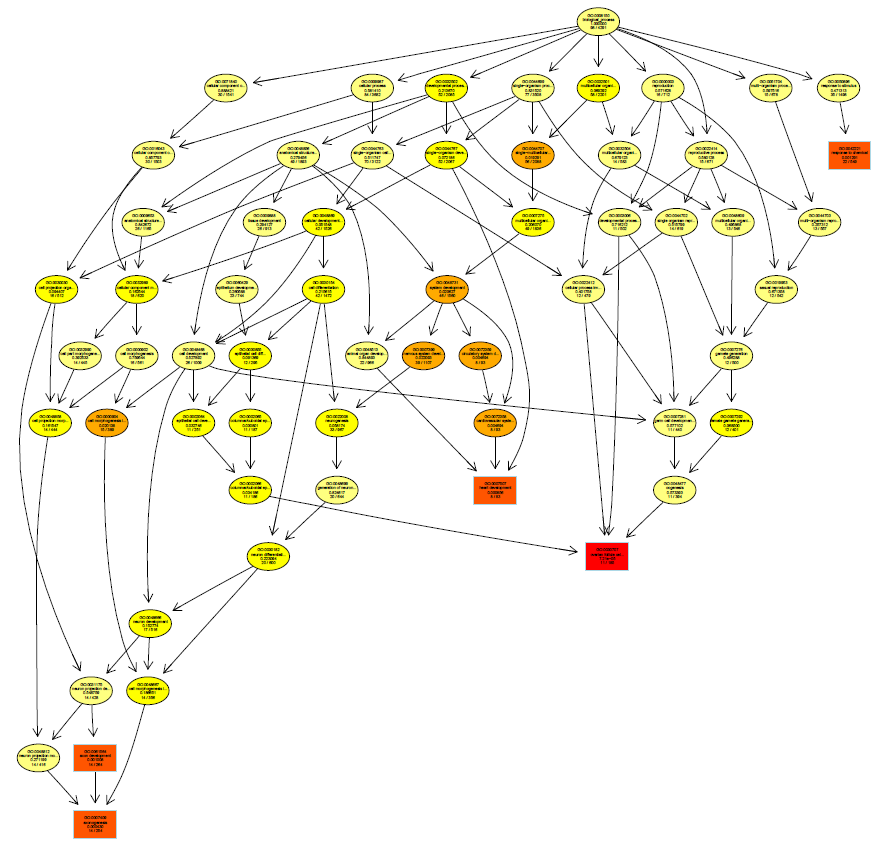


**Figure S4**: Subgraph of the top 5 GO terms identified in the gene set enrichment analysis for *S. itoensis*. Results based on Run 1 of Bayescode. Rectangles are the 5 most significant GO terms identified, with color indicating relative significance (dark red to bright yellow = most significant to least significant). Within each node the GO identifier and GO description are indicated, followed by the *p* value, and then the number of significant genes / total number of genes annotated in the dataset. The original image file is provided separately.

# References

Aranjuez G, Kudlaty E, Longworth MS, McDonald JA. 2012. On the role of PDZ domain-encoding genes in Drosophila border cell migration. G3 Bethesda Md. 2:1379–1391. doi: 10.1534/g3.112.004093.

Beall EL et al. 2002. Role for a Drosophila Myb-containing protein complex in site-specific DNA replication. Nature. 420:833–837. doi: 10.1038/nature01228.

Borghese L et al. 2006. Systematic Analysis of the Transcriptional Switch Inducing Migration of Border Cells. Dev. Cell. 10:497–508. doi: 10.1016/j.devcel.2006.02.004.

Cai D et al. 2014. Mechanical feedback through E-cadherin promotes direction sensing during collective cell migration. Cell. 157:1146–1159. doi: 10.1016/j.cell.2014.03.045.

Costa A et al. 2013. Rasputin functions as a positive regulator of orb in Drosophila oogenesis. PloS One. 8:e72864. doi: 10.1371/journal.pone.0072864.

Dobens LL, Raftery LA. 2000. Integration of epithelial patterning and morphogenesis in Drosophila ovarian follicle cells. Dev. Dyn. 218:80–93. doi: 10.1002/(SICI)1097-0177(200005)218:1<80::AID-DVDY7>3.0.CO;2-8.

Fernández-Espartero CH et al. 2013. GTP exchange factor Vav regulates guided cell migration by coupling guidance receptor signalling to local Rac activation. J. Cell Sci. 126:2285–2293. doi: 10.1242/jcs.124438.

Jang AC-C, Chang Y-C, Bai J, Montell D. 2009. Border-cell migration requires integration of spatial and temporal signals by the BTB protein Abrupt. Nat. Cell Biol. 11:569–579. doi: 10.1038/ncb1863.

Kirilly D, Spana EP, Perrimon N, Padgett RW, Xie T. 2005. BMP signaling is required for controlling somatic stem cell self-renewal in the Drosophila ovary. Dev. Cell. 9:651–662. doi: 10.1016/j.devcel.2005.09.013.

Klattenhoff C et al. 2007. Drosophila rasiRNA pathway mutations disrupt embryonic axis specification through activation of an ATR/Chk2 DNA damage response. Dev. Cell. 12:45–55. doi: 10.1016/j.devcel.2006.12.001.

Lartillot N, Poujol R. 2011. A phylogenetic model for investigating correlated evolution of substitution rates and continuous phenotypic characters. Mol. Biol. Evol. 28:729–744. doi: 10.1093/molbev/msq244.

Liu Y, Montell DJ. 2001. Jing: a downstream target of slbo required for developmental control of border cell migration. Development. 128:321–330.

Muse SV, Gaut BS. 1994. A likelihood approach for comparing synonymous and nonsynonymous nucleotide substitution rates, with application to the chloroplast genome. Mol. Biol. Evol. 11:715–724. doi: 10.1093/oxfordjournals.molbev.a040152.

Narbonne K, Besse F, Brissard-Zahraoui J, Pret A-M, Busson D. 2004. polyhomeotic is required for somatic cell proliferation and differentiation during ovarian follicle formation in Drosophila. Dev. Camb. Engl. 131:1389–1400. doi: 10.1242/dev.01003.

Perkins LA, Johnson MR, Melnick MB, Perrimon N. 1996. The nonreceptor protein tyrosine phosphatase corkscrew functions in multiple receptor tyrosine kinase pathways in Drosophila. Dev. Biol. 180:63–81. doi: 10.1006/dbio.1996.0285.

Schüpbach T, Wieschaus E. 1991. Female sterile mutations on the second chromosome of Drosophila melanogaster. II. Mutations blocking oogenesis or altering egg morphology. Genetics. 129:1119–1136.

Torres IL, López-Schier H, St Johnston D. 2003. A Notch/Delta-dependent relay mechanism establishes anterior-posterior polarity in Drosophila. Dev. Cell. 5:547–558. doi: 10.1016/s1534-5807(03)00272-7.aty E, Longworth MS, McDonald JA. 2012. On the role of PDZ domain-encoding genes in Drosophila border cell migration. G3 Bethesda Md. 2:1379–1391. doi: 10.1534/g3.112.004093.

1. Department of Bioinformatics and Genetics, Swedish Museum of Natural History [↑](#footnote-ref-2)
2. Laboratoire de Biométrie et Biologie Évolutive, University of Lyon [↑](#footnote-ref-3)
3. Vector Biology Department, Liverpool School of Tropical Medicine [↑](#footnote-ref-4)
4. Institute of Evolutionary Biology, University of Edinburgh [↑](#footnote-ref-5)
5. Biosystematics Laboratory, Faculty of Social and Cultural Studies, Kyushu University [↑](#footnote-ref-6)
6. Department of Zoology, Stockholm University [↑](#footnote-ref-7)
7. Department of Zoology, National Museum of Nature and Science, Amakubo, Tsukuba

   * Corresponding author: e-mail erik.gobbo@nrm.se [↑](#footnote-ref-8)
